# Wide excision alone for elderly patients aged > 70 years old with soft tissue sarcomas

**DOI:** 10.1097/MD.0000000000030127

**Published:** 2022-09-09

**Authors:** Yusuke Aoki, Yasunori Tome, Hiromichi Oshiro, Ryo Katsuki, Tomoko Tamaki, Naoki Wada, Kennosuke Karube, Kotaro Nishida

**Affiliations:** a Department of Orthopedic Surgery, Graduate School of Medicine, University of the Ryukyus, Okinawa, Japan; b Department of Pathology and Oncology, Graduate School of Medicine, University of the Ryukyus, Okinawa, Japan; c Department of Pathology and Laboratory Medicine, Graduate School of Medicine, Nagoya University, Nagoya, Japan.

**Keywords:** elderly, overall survival, soft tissue sarcoma, wide excision

## Abstract

The purpose of the present study was to clarify clinical outcomes of elderly patients with soft tissue sarcoma who underwent surgery neither with neoadjuvant nor adjuvant chemotherapy. The median follow-up period was 46.3 (range 6.7–99.0) months. All patients underwent surgical resections. R0 margins were achieved in 24 cases (92.3%) and R1 margins in 2 cases (7.7%). The 1-, 2-, and 5-year sarcoma-specific survival (SSS) rates were 92.3%, 88.5%, and 83.8%, respectively. Multivariate analysis showed no significant risk factors for SSS. No significant relationship of histological grades and local recurrences (*P* = .56) or distant metastases (*P* = .54) was shown. In the current study, we observed a comparable survival ratio, despite no neoadjuvant or adjuvant chemotherapies performed. Tumor resections with adequate margins might, at least in part, have contributed to the decent survival ratio regardless of histological grade. Twenty-six consecutive patients aged ≥ 70 years, who underwent surgical resections of soft tissue sarcoma between January 2013 and December 2019, were included. SSS were analyzed by the Kaplan–Meier method, and the relationships between SSS and clinical parameters were evaluated by Cox proportional hazards analysis.

## 1. Introduction

The population has been aging all over the world, especially in developed countries. In Japan, out of total population 125.22 million, 36.4 million (29.1%) are aged ≥ 65 years, which is the highest in the world.^[1,2]^

Soft tissue sarcoma (STS) is a rare malignant tumor, which is derived from mesenchymal tissues, and for which a complete surgical resection with an adequate margin with neoadjuvant and/or adjuvant chemotherapies, combination of doxorubicin and ifosfamide, is a standard therapy.^[3–5]^

Elderly patients may not be able to tolerate these aggressive chemotherapies, due to underlying diseases, complications, and decreased performance status; hence, therapeutic strategies for elderly patients need to be considered carefully.

Along with the population aging, the number of elderly patients with STS is expected to increase.^[6]^ Although some previous publications report on the management of elderly patients with STS,^[7–13]^ neoadjuvant and/or adjuvant chemotherapies were performed to some patients. The purpose of the present study was to clarify outcomes of elderly patients with STS, who underwent surgical resections without neoadjuvant nor adjuvant chemotherapies. We also examined the relationship of histological grade and survival rate, metastasis, or recurrence, since no neoadjuvant and/or adjuvant chemotherapies were performed, and therefore, it could be analyzed directly.

## 2. Materials and Methods

### 2.1. Patient selection

This was a retrospective observational study at a single institution, and included 26 consecutive patients, who aged ≥70 years and underwent surgical resection for malignant STS without neoadjuvant nor adjuvant chemotherapies, between January 2013 and December 2019. Patients with a recurrent STS, patients with distant metastases at the initial visit, patients who underwent neoadjuvant and/or adjuvant chemotherapies, and patients with <3 months follow-up were excluded from the present study. Surgical indications were carefully judged, based on the patient’s life expectancy, general condition, and concurrent comorbidities, which can affect the patient’s survival. The present study was approved by the Institutional Review Boards of our institution (no. 1665). Written informed consent was obtained from all the patients before study enrollment.

### 2.2. Parameters

Medical charts were retrospectively reviewed and patient’s information, including age, gender, tumor size, tumor depth, location of the tumors, surgical procedure, histological diagnosis, surgical margin, radiation therapy, histological grade according to Fédération Nationale des Centres de Lutte Contre Le Cancer (FNCLCC) classification,^[14]^ postoperative complication, postoperative local recurrence and distal metastasis, postoperative follow-up time, and outcomes, was collected. Size and location of tumors were estimated using magnetic resonance imaging and/or computed tomography, and size was defined as the maximum diameter. Tumor size was classified as ≤5 cm, or >5 cm and ≤10 cm, or >10 cm. Tumor depth was dependent on whether the tumors developed deeper than the superficial fascia or not. Surgical margins were classified as previously reported.^[15]^ Postoperative complications included surgical-site infection and delayed wound healing.

### 2.3. Therapeutic strategy

All 26 enrolled patients underwent surgical resection without neoadjuvant nor adjuvant chemotherapy. Firstly, wide excision was planned as long as possible, but in case the tumor was adjacent to critical nerves and/or vessels, a wide excision, using the in situ preparation method,^[16]^ was planned. In case critical nerves and/or vessels were involved by the tumor, amputation was planned eventually. Surgical resections were aimed to guarantee adequate margins from tumor tissue for all cases, as far as possible.

### 2.4. Statistical analysis

Survival analyses were performed using the Kaplan–Meier method. Sarcoma-specific survival (SSS) was defined as from the date of surgery to the date of sarcoma-related death or the last follow-up for survivors. Fishers exact test was used to compare between 2 categorical variables. Cox proportional hazards analysis was used to reveal factors associated with SSS. All statistical analyses were performed using JMP software version 15 (SAS Institute Inc., Tokyo, Japan). Data were reported with hazard ratio (HR), 2-sided 95% confidence intervals (CIs), and *P* values. A *P* value ≤.05 was defined as statistically significant difference.

## 3. Results

### 3.1. Patients and tumor characteristics

A total of 26 patients who underwent surgical resection without neoadjuvant nor adjuvant chemotherapy were enrolled in this study. The median follow-up period was 46.3 (range 6.7–99.0) months. The median age at the initial visit was 80.5 (range 70–91) years (70–74 years old: 7 patients, 75–79 years old: 5 patients, ≥80 years old: 14 patients). The size of tumors were as follows: ≤5 cm in 11 patients, >5 cm or ≤10 cm in 9 patients, >10 cm in 6 patients. The primary tumors in 13 patients were in superficial layer, which is shallower than the superficial fascia, and the tumors in 13 patients developed deeper than the superficial fascia. The sites of the primary tumors were as follows: 4 in the upper extremities, 17 in the lower extremities, and 5 in the trunk of the body. Histological diagnoses were as follows: myxofibrosarcoma in 11 patients, undifferentiated pleomorphic sarcoma in 7 patients, leiomyosarcoma in 4 patients, liposarcoma in 3 patients, and malignant peripheral nerve sheath tumor (MPNST) in a patient. Twenty-four patients underwent wide excision alone and 2 patients underwent wide excision using the in situ *preparation* method.^[16]^ R0 margins were achieved in 24 patients (92.3%) and R1 in 2 patients (7.7%). Adjuvant radiation therapy was performed for 2 patients with R1 margin. Based on the Fédération Nationale des Centres de Lutte Contre Le Cancer classification, 6 patients were classified as grade 1, 11 patients were classified as grade 2, 9 patients were classified as grade 3. Delayed wound healing occurred in 2 patients, and wound infection occurred in a patient (Table 1).

**Table 1:** Patients and tumors characteristics.

	N
Age	
Median (range)	80.5 (70–91)
70–74 yr	7
75–79 yr	5
≥80 yr	14
Sex	
Male	16
Female	10
Size (cm)	
<5	11
≥5 and <10	9
≥10	6
Depth	
Superficial	13
Deep	13
Location	
Upper extremity	4
Lower extremity	17
Trunk	5
Histological diagnosis	
Myxofibrosarcoma	11
UPS	7
Leiomyosarcoma	4
Liposarcoma	3
Others	1
Surgical margin	
R0	24
R1	2
Adjuvant radiation therapy	
+	2
−	24
FNCLCC	
Grade 1	6
Grade 2	11
Grade 3	9
Complication	
Delayed wound healing	2
Infection	1

FNCLCC = Fédération Nationale des Centres de Lutte Contre Le Cancer, UPS = undifferentiated pleomorphic sarcoma.

### 3.2. Oncological outcome and postoperative result

Oncological outcomes at the last follow-up were as follows: continuously disease-free in 20 patients, alive with disease in 2 patients, and died of disease (DOD) in 4 patients (Table 2A). Postoperative local recurrences occurred in 5 patients (19.2%). Four out of these 5 patients underwent rewide excision and the rest 1 patient received radiation therapy alone. Three out of these 4 patients who underwent rewide excision were alive at the last follow-up without recurrence nor metastasis, while the rest 1 patient eventually resulted in DOD. Distant metastases postoperatively occurred in 4 patients (15.4%). Three out of these 4 patients had metastases in the lung and eventually DOD. The rest 1 patient, who had metastasis in the clavicle, received palliative chemotherapy, including pazopanib and cyclophosphamide, and was alive with disease at the last follow-up (Table 2B).

**Table 2A:** Oncological outcome.

	N
Oncological outcome	
CDF	20
AWD	2
DOD	4

AWD = alive with disease, CDF = continuously disease-free, DOD = dead of disease.

**Table 2B:** Postoperative local recurrences and distant metastases.

	N	Additional therapy	N	Outcome	N
Local recurrence	5	Rewide excision	4	CDF	3
				DOD	1
		Radiation therapy	1	AWD	1
Distant metastasis	4	Best supportive care	3	DOD	3
		Palliative chemotherapy	1	AWD	1

AWD = alive with disease, CDF = continuously disease-free, DOD = dead of disease.

### 3.3. Survival rates and clinical factors

**Figure 1.**
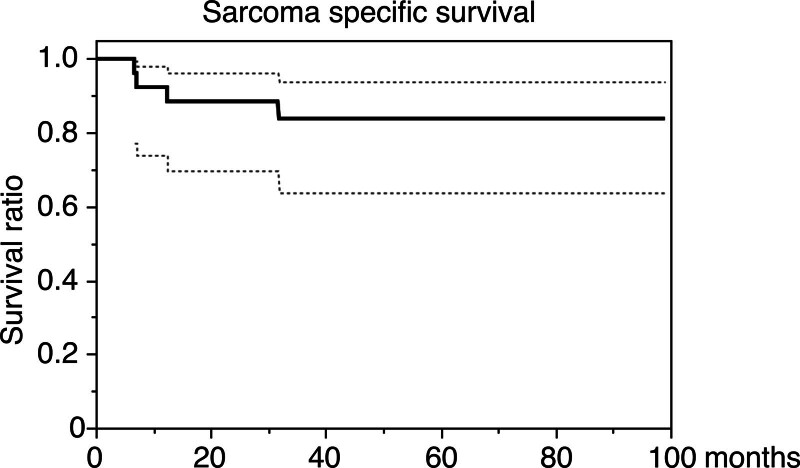
Kaplan–Meier curve showing sarcoma-specific survival rate of 26 elderly patients with soft tissue sarcoma after wide excision.

The 1-, 2-, and 5-year SSS rates were 92.3%, 88.5%, and 83.8%, respectively (Fig. 1). According to Fisher exact test, there was no significant relationship of histological grades and local recurrences (*P* = .56) or distant metastases (*P* = .54; Tables 3A, B).

**Table 3A:** The relationship between histological grade and local recurrence.

	Low grade	High grade	Total	*P* value
Recurrence				
+	2	3	5	
−	4	17	21	
Total	6	20	26	.56

**Table 3B:** The relationship between histological grade and distant metastasis.

	Low grade	High grade	Total	*P* value
Metastasis				
+	0	4	4	
−	6	16	22	
Total	6	20	26	.54

Multivariate analysis by Cox proportional hazards model showed no significant risk factors for SSS as follows: age (hazard ratio [HR] = 0.70, 95% CI = 0.17–3.05, *P* = .62), histological grade (HR = 0.35, 95% CI = 0.09–1.42, *P* = .14), local recurrence (HR = 3.21, 95% CI = 0.70–14.7, *P* = .13), distal metastasis (HR = 0.89, 95% CI = 0.11–7.03, *P* = .91; Table 4).

**Table 4:** Multivariate Cox proportional hazards models of overall survival.

	HR	95% CI	*P* value
Age	0.70	0.17–3.05	.62
Grade	0.35	0.09–1.42	.14
Local recurrence	3.21	0.70–14.7	.13
Distal metastasis	0.89	0.11–7.03	.91

CI = confidence interval, HR = hazard ratio.

## 4. Discussion

The 1-, 2-, and 5-year SSS rates were 92.3%, 88.5%, and 83.8%, which are comparable to previous reports,^[7–13]^ despite neither neoadjuvant nor adjuvant chemotherapies performed. Although chemotherapies could decrease micrometastases and residual tumors, especially the combination therapy of doxorubicin and ifosfamide has been reported to be effective for STS patients who underwent surgical resection,^[3–5]^ elderly patients are generally vulnerable to chemotherapies; hence, indication of chemotherapy must be judged carefully. The result of the present study could suggest that, for elderly patients, surgical resections without neoadjuvant nor adjuvant chemotherapies might be reasonable.

Since neoadjuvant/adjuvant chemotherapies were not performed in the present study, the relationship of histological grade and SSS, metastasis, or recurrence could be analyzed directly. There was no relationship of histological grade and local recurrence or distant metastasis, which is inconsistent to previous reports that high histological grade is a highly significant independent risk factor for survival ratio.^[7,8]^

In the present study, R0 margins were achieved in 24 out of 26 patients (92.3%). As for the remaining 2 patients, in whom R1 margins were observed, 1 patient had distant metastases 7 months after surgery and eventually DOD, and the other patient had a local recurrence 1 year after surgery and underwent reexcision and was alive with continuously disease-free at the last follow-up. Tumor resections with adequate margins in most patients might, at least in part, have contributed to the decent SSS regardless of histological grade, which is consistent to previous reports that surgical margins significantly contribute to survival.^[8–10]^

There are several limitations in the current study. The first is that the study was a retrospective review of records with a small sample size at a single institution. STS is not a common type of tumor, and furthermore, in the present study, participants were limited to patients with STS aged ≥ 70 years, who underwent surgical resections without neoadjuvant nor adjuvant chemotherapies. The second is that the study included only patients who had undergone surgical resection, which may have led to some selection bias, because all the included patients were judged to be able to tolerate operations, excluding patients who were unable to undergo surgeries. The third is that the effect of neoadjuvant/adjuvant chemotherapy was not discussed or compared. In the future study, we will conduct joint research with other institutions to collect more patients and compare the effect of chemotherapy for elderly patients with STS.

## 5. Conclusion

In the current study, we observed a comparable survival ratio, despite no neoadjuvant or adjuvant chemotherapies performed. Tumor resections with adequate margins in most included patients might, at least in part, have contributed to the decent SSS regardless of histological grade. This hypothesis needs to be validated with additional studies in the future.

## Author contributions

All authors have read and agreed to the published version of the manuscript.

**Conceptualization:** Yusuke Aoki, Yasunori Tome.

**Data curation:** Yusuke Aoki, Yasunori Tome, Ryo Katsuki, Tomoko Tamaki, Naoki Wada, Kennosuke Karube.

**Formal analysis:** Yusuke Aoki.

**Funding acquisition:** Kotaro Nishida.

**Investigation:** Yusuke Aoki, Yasunori Tome, Hiromichi Oshiro, Ryo Katsuki.

**Methodology:** Yusuke Aoki, Yasunori Tome, Hiromichi Oshiro.

**Project administration:** Yusuke Aoki, Yasunori Tome, Hiromichi Oshiro, Kotaro Nishida.

**Resources:** Yasunori Tome, Hiromichi Oshiro, Tomoko Tamaki, Naoki Wada, Kennosuke Karube.

**Software:** Yusuke Aoki.

**Supervision:** Yasunori Tome, Kotaro Nishida.

**Validation:** Yusuke Aoki, Yasunori Tome.

**Visualization:** Yusuke Aoki.

**Writing – original draft:** Yusuke Aoki, Yasunori Tome, Kotaro Nishida.

**Writing – review & editing:** Yasunori Tome, Kotaro Nishida.
